# Dengue Encephalopathy or Dengue Encephalitis? You Decide

**DOI:** 10.1093/ofid/ofad490

**Published:** 2023-10-03

**Authors:** Simon M Durkin, Andrea L da Silva, Nicholas W S Davies, Shiranee Sriskandan

**Affiliations:** Department of Clinical Infectious Diseases, Hammersmith Hospital, Imperial College Healthcare National Health Service Trust, London, United Kingdom; Department of Radiology, Charing Cross Hospital, Imperial College Healthcare National Health Service Trust, London, United Kingdom; Department of Neurology, Chelsea and Westminster Hospital, Chelsea and Westminster Hospitals National Health Service Foundation Trust, London, United Kingdom; Department of Clinical Infectious Diseases, Hammersmith Hospital, Imperial College Healthcare National Health Service Trust, London, United Kingdom; Department of Infectious Disease, Faculty of Medicine, Imperial College London, Hammersmith Hospital, London, United Kingdom

**Keywords:** brain diseases, dengue, encephalitis, severe dengue, viral

## Abstract

Awareness of neurological sequelae of dengue fever is increasing. However, as this case illustrates, there is a diagnostic conundrum in determining whether certain features are in keeping with dengue encephalopathy or dengue encephalitis. Further consensus is required.

Dengue fever, the most rapidly spreading mosquito-borne viral illness, caused by dengue virus (DENV) serotypes 1–4, generates a significant burden of morbidity as well as socioeconomic impacts. There is increasing awareness of neurological sequelae: Between 0.5% and 21% of patients with dengue fever exhibit neurological symptoms and signs [1]. The World Health Organization (WHO) reclassified dengue syndromes in 2009, incorporating neurological signs as a marker of severe dengue [2]. Here, we present a case that highlights the diagnostic challenges in classification of encephalopathy versus encephalitis in dengue.

## CASE REPORT

A 50-year-old female fitness instructor and domestic worker with a history of amlodipine-controlled hypertension presented to a teaching hospital in the United Kingdom (UK) with a 2-day history of fever and myalgia with prostration, accompanied by a worsening headache and photophobia. The patient was originally from the Philippines but had lived for many years in the UK with her family. She had traveled for 10 days to Manila, visiting relatives and staying outside the city in the suburbs. She returned to the UK 5 days prior to admission. Although she had taken precautions, she was bitten by mosquitoes; she noted that a number of friends and relatives had also been hospitalized in the Philippines with dengue fever.

Initial cardiorespiratory and neurological examination was normal with no focal neurological signs, including meningism. No mucosal bleeding or skin rash was seen. Ceftriaxone and acyclovir were commenced as empirical treatment for meningitis and encephalitis. Three blood films and 3 rapid diagnostic tests were negative for malaria parasites. Blood and urine cultures demonstrated no growth. Computed tomographic (CT) brain scan showed no acute intracerebral pathology, with normal grey–white matter differentiation. A lumbar puncture was performed within 24 hours of admission (day 3 of symptoms); platelet count was 239 × 10^9^/L. Cerebrospinal fluid (CSF) was acellular (white blood cells <1/μL, red blood cells <1/μL), with no organisms seen on microscopy and no growth on bacterial culture. A routine CSF virology polymerase chain reaction (PCR) screening panel (herpes simplex virus, varicella zoster virus, enterovirus) was negative and therefore acyclovir was stopped. An extended-panel respiratory virus PCR throat swab (Supplementary Table 1) was negative. Serology for human immunodeficiency virus types 1 and 2, hepatitis B virus, hepatitis C virus, and *Borrelia burgdorferi* infections were negative. Serology for both Epstein-Barr virus and cytomegalovirus showed evidence of previous infection.

A serum sample sent to the reference laboratory for analysis demonstrated immunoglobulin M (IgM) and immunoglobulin G, as well as PCR, positivity for DENV. Serotyping was not performed. Subsequent PCR testing of CSF demonstrated positivity for DENV RNA. Full details of all tests performed are outlined in Supplementary Table 1.

The patient was transferred to the infectious diseases ward for ongoing management. She developed coagulopathy and abnormal liver function tests (transaminitis) midway through her inpatient stay. Ferritin was maximally 48 000 μg/L (range, 10–120 μg/L) with alanine aminotransferase 350 U/L (range, 0–34 U/L), activated partial thromboplastin time (APTT) 75 seconds (range, 25–35 seconds), and platelet nadir 35 × 10^9^/L (range, 135–400 × 10^9^/L).

**Figure 1.**
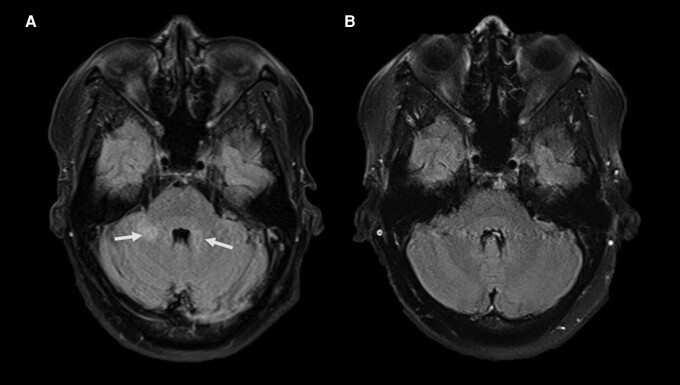
*A*, Axial magnetic resonance imaging fluid-attenuated inversion recovery (FLAIR) image, day 7 of illness, demonstrating ill-defined areas of T2-weighted signal hyperintensity in both cerebral peduncles (arrows). *B*, Axial FLAIR image 15 months later demonstrating resolution of the abnormal signal in the cerebral peduncles.

On day 6 of the admission (day 8 of illness), the patient had an unwitnessed fall without head injury after dizziness on mobilizing to the toilet. She was noted to be less alert with a Glasgow Coma Scale (GCS) score of 13 (E4, M5, V4) and was unable to obey commands. She was observed to have intermittent vacant episodes and confused speech with word-finding difficulties. Given concurrent thrombocytopenia, further cerebral imaging was obtained. A repeat CT brain scan showed no acute intracranial pathology. Consequently, a magnetic resonance imaging (MRI) brain scan was performed (Figure 1), which showed an ill-defined rounded T2-dependent signal abnormality, without diffusion restriction, in both the left and right middle cerebellar peduncles (the latter being more affected), suggestive of an underlying inflammatory process. There were also multiple small nonspecific scattered supratentorial, subcortical, and deep white matter T2-hyperintense foci, most likely representing age-appropriate cerebral microangiopathic change.

Her level of coherence and mentation as well as her balance and dizziness normalized spontaneously during the remainder of her stay. All blood parameters improved spontaneously—platelets were normal and APTT was 48 seconds—by discharge. She was discharged on day 11 of admission. The patient re-presented to the emergency department 14 days after this admission with a medication overuse headache secondary to codeine phosphate for post-dengue myalgia and headache. Neurological examination including fundoscopy was normal. CT brain scan at this time was normal with no acute intracranial pathology; MRI was not performed. Symptoms resolved spontaneously on cessation of codeine.

Outpatient interval MRI brain was postponed due to the coronavirus disease 2019 pandemic but occurred at 15 months post-admission. There was near-complete resolution of the cerebellar changes, temporally related to resolution of the dengue infection. A review of the initial diffusion-weighted imaging sequences did not show any associated microhemorrhage. There remained a 2-mm siderotic nodule in the region of the right cerebellar peduncle abnormality, likely representing a small fleck of calcification and consistent with healing of an infective or inflammatory process.

## DISCUSSION

In 2009, the WHO reclassified DENV clinical cases, incorporating neurological signs and symptoms as a manifestation of severe dengue [2]. In one review, neurological complications occurred in 0.5%–5.4% of confirmed dengue cases originating in southeast Asia [3]; in other studies, up to 20% of cases of encephalitis presenting at hospitals were diagnosed with dengue [1].

In the above case, impairment of consciousness (“severe organ involvement”) categorizes the patient as having severe dengue. Currently, no clear diagnostic criteria exist for distinguishing dengue encephalopathy from encephalitis, and the terms are often used interchangeably [1]. There have long been calls for standardized criteria to define these conditions in dengue [1, 3, 4]. A classification of dengue involvement of the CNS has been proposed recently [1], but there is debate as to the possibility of underdiagnosis [4].

Encephalopathy, an altered conscious level, is the most commonly reported neurological complication of dengue, ordinarily exhibiting normal CSF cell counts; however, in a number of reported case series, no PCR testing of CSF for DENV was performed [1]. Dengue encephalopathy may result from shock, cerebral edema, electrolyte abnormality, acute liver or kidney injury, or cerebrovascular complications such as microhemorrhages [1].

While not previously thought to be the case, it is now known that DENV, like other flaviviruses, exhibits tropism for neural cells, including direct neuroinvasion [5]. In our case, DENV RNA was concomitantly detected in blood, but previous cases where CSF PCR alone was positive again suggest active neuroinvasion, rather than vascular leakage or permeation across the blood–brain barrier [1]. In some instances of encephalitis, a lymphocytic pleocytosis may add weight to the diagnosis, but in others CSF cellularity is reported to be normal [6], as here, and this does not exclude viral encephalitis [1, 5]. Timing of the lumbar puncture in the course of the illness may have affected this result. Brain imaging may show heterogeneous changes, or appear normal, in dengue encephalitis cases [1, 5, 6]. Dengue cerebellitis has also been described [7].

In this case, there was evidence of acute dengue infection (serum IgM positivity, epidemiological risk), dengue central nervous system (CNS) involvement (altered GCS), and evidence of DENV (positive RNA PCR) in CSF that was otherwise acellular (and therefore unlikely to be passively contaminated with DENV RNA from a traumatic tap). Although present, the degree of liver function abnormality was not sufficient to explain the patient's symptoms. However, there was no CSF pleocytosis. The brain lesions found on MRI are nonspecific but temporally were associated with the initial infection, appear inflammatory in nature, and improved to resolution on subsequent imaging. Electroencephalography was not performed in this case; nor was there evidence of seizure activity, focal or generalized, in contrast to other cases of CNS dengue classified as encephalitis [3]. Histopathology demonstrating viral inclusions with inflammation and/or necrosis might have been declarative, but sampling in this case was not undertaken.

In summary, there is no clear expert consensus on classification of encephalopathy versus encephalitis syndromes in dengue infection. Differentiation between dengue encephalopathy and encephalitis will be of increasing importance, especially with increasing dengue prevalence. Studies have shown an increased risk of encephalitis in secondary (compared with primary) dengue, as well as higher mortality rates in cases classified as dengue encephalitis [8]. One may presume that patients suffering from encephalitis might have a higher burden of long-term neurological and other sequelae than those with encephalopathy, but further research on this would be required. Formulation of consensus criteria would therefore aid identification of dengue severity, standardize workup, and assist with stratified prognostication and early treatment planning (such as transfer to an inpatient or secondary/tertiary center), as well as providing another benchmark against which to test vaccine candidates. In a similar way, dengue must be considered in patients presenting with encephalitis, to ensure prompt diagnosis of dengue.

This case fulfills the proposed neurological Koch postulates for viral etiologies of encephalitis [9]. In the absence of any other discernible cause, this case could be classified as probable or confirmed dengue encephalitis by some criteria [4, 9]—a diagnosis that may not be as rare as previously thought. Disagreement exists in particular as to inclusion of CSF pleocytosis and imaging changes, and, as discussed, their prevalence in dengue encephalitis cases is variable. Further global expert consensus is therefore required to clarify the dengue encephalopathy versus dengue encephalitis distinction.

## Supplementary Data

Supplementary materials are available at *Open Forum Infectious Diseases* online. Consisting of data provided by the authors to benefit the reader, the posted materials are not copyedited and are the sole responsibility of the authors, so questions or comments should be addressed to the corresponding author.

## Supplementary Material

ofad490_Supplementary_Data

## Notes

***Author contributions.*** S. M. D.: conceptualization, writing–original draft preparation, writing–review and editing, patient care. A. L. d. S.: writing–original draft preparation, patient care (imaging review). N. W. S. D.: writing–review and editing, patient care (specialist review). S. S.: writing–review and editing, supervision, patient care (named consultant).

***Acknowledgments.*** The authors are grateful for the kind permission of the patient to publish this case. The authors also thank Dr Jane Osborne, Consultant Clinical Scientist, and the team at the Rare and Imported Pathogens Laboratory, Porton Down, United Kingdom, for their involvement in processing and interpreting samples.

***Patient consent.*** The patient provided written informed consent to publish this case report.
