# Supplementary material for: Dengue Encephalopathy or Dengue Encephalitis? You Decide
Source: Open Forum Infect Dis. 2023 Oct 3;10(10):ofad490. doi: 10.1093/ofid/ofad490 (PMC10586725; doi:10.1093/ofid/ofad490)
Supplement: ofad490_Supplementary_Data [file ofad490_supplementary_data.docx]

**Supplementary Table 1.** Summary of microbiological investigations performed – CSF, serum, and other.

| **Investigation** | **Result** |
| --- | --- |
| **CSF**  WBC  RBC  Microscopy  Culture  Protein  Glucose  Lactate dehydrogenase  Lactate  Xanthochromia  **CSF virology**  Dengue RNA*  (AusDiagnostics CNS Pathogens (12-well), High-Plex 24 System)  HSV-1  HSV-2  Varicella Zoster virus  Adenovirus  Cytomegalovirus  Epstein-Barr virus  Enterovirus  Parechovirus  HHV-6  **Serum virology**  Dengue RNA PCR*  Dengue IgM†  Dengue IgG†  Zikavirus NS1 RNA PCR  Chikungunya RNA PCR  Leptospira Lip32 & 16S DNA PCR  Tick borne encephalitis virus IgG (EIA)  Zikavirus IgM (EIA)  Zikavirus IgG (EIA)  Chikungunya IgM (EIA)  Chikungunya IgG (EIA)  **Extended-panel respiratory viral PCR throat swab**  (AusDiagnostics Respiratory Pathogens (16-well), High-Plex 24 System)  Influenza A  Influenza B  Respiratory syncytial virus  Parainfluenza 1/2/3/4  Adenovirus  Human metapneumovirus  *Bordetella pertussis*  *Mycoplasma pneumoniae*  **Routine microbiology**  Blood cultures  Urine culture  HIV-1/-2 serology  Hepatitis B & C serology (serum)  Malaria rapid diagnostic test (x2)  Syphilis serology (serum)  EBV serology (serum)  CMV serology (serum)  *Borrelia burgdorferi* (Lyme) serology (serum) | <1/uL  <1/uL  No organisms  No growth  0.32g/L (range 0.15-0.45g/L)  3.3mmol/L  <30 IU/L  1.7mmol/L  Negative  Positive  Negative  Negative  Negative  Negative  Negative  Negative  Negative  Negative  Negative  Positive  Positive  Negative  Negative  Negative  Negative  Positive (>1:10,000) (*likely cross-reaction)  Negative  Negative  Negative  Negative  Negative  Negative  Negative  Negative  Negative  Negative  Negative  Negative  No growth  No growth  Negative  Negative  Negative  Negative  IgG positive, IgM negative (*past infection)  IgG positive, IgM negative (*past infection)  Negative |

* Dengue PCR: Performed on an in-house DENV serotypes 1-3 real-time PCR assay at RIPL. Ct values: CSF = 29.9, blood = 24.0

† Dengue serology: Performed on the Panbio IgG/IgM commercial assay at RIPL.
